# First-in-human and multicenter phase I study of OSCA therapy for knee osteoarthritis

**DOI:** 10.1038/s12276-026-01728-w

**Published:** 2026-05-01

**Authors:** Dae Keun Suh, Sang Hak Lee, Yohan Bae, Hee-Jin Kang, Seunghee Lee, Mijin Kim, JongCheon Na, Ali Mobasheri, Kyung-Sun Kang, Kyoung Ho Yoon

**Affiliations:** 1 Department of Orthopaedic Surgery, Kyung Hee University Hospital 23, Seoul, Republic of Korea; 2 Department of Orthopaedic Surgery, Kyung Hee University Hospital at Gangdong, Seoul, Republic of Korea; 3 Kangstem Biotech Co., Ltd, Hongwoo Building, Seoul, Republic of Korea; 4 Stem Cell and Regenerative Bioengineering Institute, Global R&D Center, Kangstem Biotech Co. Ltd, Seoul, Republic of Korea; 5 Research Unit of Health Sciences and Technology, Faculty of Medicine, University of Oulu, Oulu, Finland; 6 Department of Personalized Medicine, State Research Institute Centre for Innovative Medicine, Vilnius, Lithuania; 7 Department of Joint Surgery, First Affiliated Hospital of Sun Yat-sen University, Guangzhou, Guangdong Province China; 8 Faculté de Médecine, Université de Liège, Quartier Hôpital — Avenue Hippocrate 15 (Bât. B36), Liège, Belgium; 9 Adult Stem Cell Research Center, and Research Institute for Veterinary Science, College of Veterinary Medicine, Seoul National University, Seoul, Republic of Korea

**Keywords:** Stem-cell research, Mesenchymal stem cells, Stem-cell differentiation

## Abstract

Knee osteoarthritis is a degenerative joint disease, with no fundamental cure beyond pain relief and anti-inflammation. This phase I, multicenter, open-label, dose-escalation trial evaluated the safety, tolerability, and preliminary efficacy of OSCA, an intra-articular injection co-formulation of osiramestrocel (allogeneic mesenchymal stromal cells) and cartilage acellular matrix, in patients with Kellgren–Lawrence (K&L) grade 2 or 3 knee osteoarthritis. Twelve patients received a single intra-articular injection of OSCA at low (2.5 × 10^7^ cells, *n* = 3), mid (5.0 × 10^7^ cells, *n* = 3), or high (1.0 × 10^8^ cells, *n* = 6) doses; cell concentration was 3.3 × 10^7^ cells/ml for all cohorts, and cartilage acellular matrix was 60 mg (40 mg/ml). The primary end point was safety, assessed by dose-limiting toxicities and TEAEs. Secondary end points included Visual Analog Scale (VAS), International Knee Documentation Committee (IKDC), Magnetic Resonance Observation of Cartilage Repair Tissue (MOCART), Whole-Organ Magnetic Resonance Imaging Score (WORMS), and K&L grading over 24 weeks. OSCA was well tolerated, with no dose-limiting toxicities and mild-to-moderate TEAEs in 25.0% (3/12) of patients. At 24 weeks, mid-dose and high-dose groups showed numerically greater improvement in pain and function than the low-dose group (*P* < 0.05), with up to 91.3% reduction in VAS (*P* = 0.002) and 102.7% IKDC (*P* = 0.005). MRI outcomes showed efficacy signals: MOCART total scores improved in 70% of patients, WORMS cartilage integrity improved in 50%, and K&L grades remained stable in 80%. Overall, these findings support the feasibility and tolerability of a single OSCA injection, with exploratory efficacy in both clinical and MRI outcomes.

## OSCA injection shows promise for knee osteoarthritis relief

Osteoarthritis, a prevalent degenerative joint disease, lacks effective treatments that modify its progression or restore joint function. This study introduces OSCA, a novel therapy combining human umbilical cord blood-derived mesenchymal stromal cells with cartilage acellular matrix, aiming to enhance cartilage regeneration in knee osteoarthritis. Conducted as a first-in-human, multicenter, open-label clinical trial, the study evaluated the safety, tolerability, and potential disease-modifying effects of OSCA through intra-articular injections in 12 patients. Results showed that OSCA was well tolerated, with no dose-limiting toxicities, and demonstrated promising improvements in pain and function scores, particularly in higher dose cohorts. MRI assessments indicated potential cartilage repair and joint preservation, although osteophyte progression was noted in some cases. These findings suggest OSCA’s potential as a disease-modifying osteoarthritis drug, warranting further investigation in larger, long-term trials to confirm its efficacy and safety. This summary was initially drafted using artificial intelligence, then revised and fact-checked by the author.

## Introduction

Osteoarthritis is a degenerative joint disease characterized by progressive cartilage loss, subchondral bone remodeling, synovial inflammation, and joint pain, ultimately leading to functional impairment and disability^1,2^. Osteoarthritis, a leading cause of chronic pain and disability worldwide, sustantially impairs the quality of life of patients and imposes a major socioeconomic burden^3,4^. Despite its prevalence, osteoarthritis still lacks treatments that modify disease progression or restore joint function. Current therapies — analgesics, non-steroidal anti-inflammatory drugs, and intra-articular (IA) injections — mainly target symptomatic relief, as no disease-modifying osteoarthritis drug (DMOAD) has been approved^5–7^.

Recently, mesenchymal stem cell (MSC)-based therapies have demonstrated potential for cartilage protection and regeneration in osteoarthritis^8–12^. However, structural recovery in the knee joint, such as cartilage repair and subchondral bone regions, has not been consistently demonstrated in clinical studies. Among them, studies using MSCs have shown either no meaningful changes in bone marrow lesions or cartilage morphology, or only limited evidence of cartilage regeneration in short-term follow-up^13–15^. Among various MSC sources, human umbilical cord blood-derived MSCs stand out as a promising regenerative therapy for osteoarthritis owing to their high proliferative capacity, low immunogenicity, and secretion of bioactive factors that facilitate cartilage repair and modulate the joint microenvironment^11,12,16^. Therefore, to date, no approved DMOAD effectively stops disease progression, preserves joint structure, and improves physical function beyond pain relief.

OSCA is a novel therapy, a co-formulation of OSiramestrocel (human umbilical cord blood-derived allogeneic mesenchymal stromal cells) and Cartilage Acellular matrix (CAM) that enhances cartilage regeneration through their synergistic interaction. As small animal models such as rabbits and rats have excellent self-healing ability, it is difficult to expect equivalent efficacy in actual clinical practice^17,18^. Therefore, for the clinical application, it is required to evaluate the therapeutic efficacy, stability, and biological safety in large animal models that have less self-healing ability and better mimic the anatomical features of human articular joints^19–21^. Our previous study demonstrated that IA OSCA injections sustantially promoted cartilage repair, modulated the joint microenvironment, reduced inflammation, and supported subchondral bone remodeling, which highlighted their potential as a DMOAD^22,23^. Therefore, this first-in-human phase I study aims to evaluate the safety and tolerability of OSCA and its potential disease-modifying effects on joint structure in patients with knee osteoarthritis.

## Methods

### Study design and patient eligibility

This study is a first-in-human, multicenter, open-label clinical trial designed to evaluate the safety, tolerability, and clinical efficacy of OSCA. Patient enrollment began on July 11, 2023, and the last patient visit was conducted on August 6, 2024. The clinical trial adhered to the guidelines of the Declaration of Helsinki and was approved by the Institutional Review Board (IRB) of Kyung Hee University Hospital and Kyung Hee University Hospital at Gangdong (IRB no. 2023-01-073-004 and 2023-01-008) and registered at ClinicalTrials.gov (NCT05944627). Written informed consent was obtained from all patients before enrollment.

Eligible patients were adults ($\ge$19 years old) diagnosed with knee osteoarthritis based on standard weight-bearing radiographs showing Kellgren–Lawrence (K&L) grade 2–3 and confirmed by the International Cartilage Repair Society (ICRS) grade 3 or 4 rating system through MRI examination at the time of screening. Additionally, participants were required to have a Visual Analog Scale (VAS) pain score of $\ge$50 mm on a 100-mm scale at screening and persistent symptoms (pain, and so on) that do not improve after at least 12 weeks of conservative treatment (drug therapy, physical therapy, and so on). Detailed inclusion and exclusion criteria are provided (Supplementary Table 1).

### Enrollment and study protocol

**Fig. 1:**
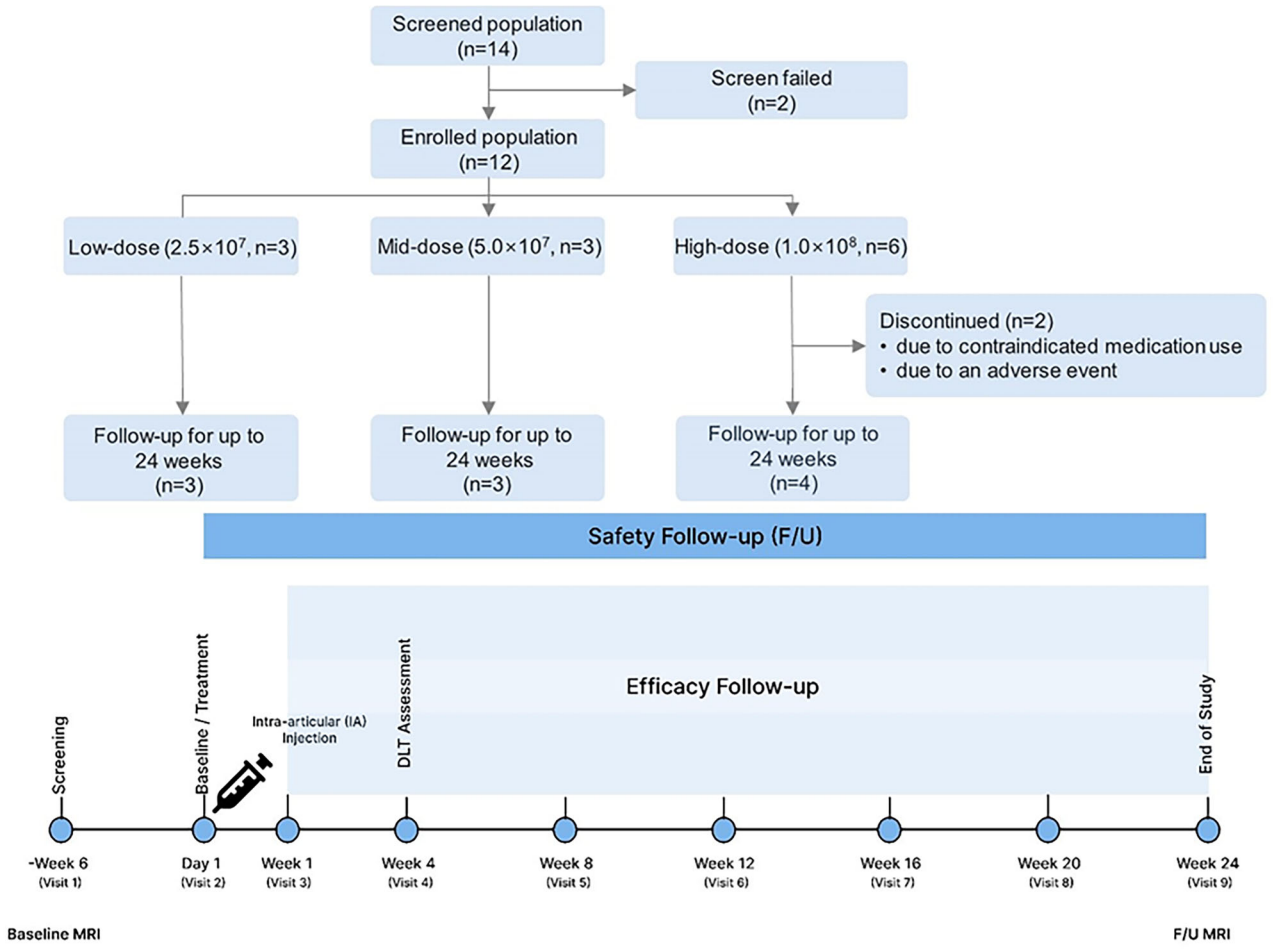
Details of study protocol and flow diagram. This figure illustrates the study design, including participant allocation, follow-up schedule, and assessment timeline. Fourteen patients with knee osteoarthritis were screened and allocated to three cohorts: low-dose (*n* = 3), middle-dose (*n* = 3), and high-dose (*n* = 6). Two patients from the high-dose cohort discontinued participation owing to contraindicated medication use (*n* = 1) and adverse events (*n* = 1). All remaining participants completed follow-up assessments over 24 weeks. Assessments were conducted on day 1 and at weeks 1, 4, 8, 12, 16, 20, and 24 post-intra-articular OSCA administration to assess safety and exploratory efficacy via pain and function scores. Radiological exploratory end points were evaluated at baseline and at 24 weeks, whereas biomarker analyses were conducted at baseline and weeks 1, 4, 12, and 24. DLT, dose-limiting toxicity.

Following eligibility assessment, patients were sequentially enrolled and administered a single IA injection of OSCA at one of three escalating doses. A single IA injection containing 2.5 $\times$ 10^7^ cells (cohort 1, low-dose), 5.0 $\times$ 10^7^ cells (cohort 2, mid-dose), or 1.0 $\times$ 10^8^ cells (cohort 3, high-dose), with a cell concentration of 3.3 $\times$ 10^7^ cells/ml for all cohorts, combined with CAM 60 mg at a concentration of 40 mg/ml, was administered. Dose escalation followed a standard 3 $+$ 3 design based on dose-limiting toxicities (DLTs). Patients were followed up on day 1 and weeks 1, 4, 8, 12, 16, 20, and 24 post-injection to assess safety and exploratory efficacy using pain and functional scores. Additionally, radiological end points were evaluated at baseline and 24 weeks (Fig. 1). During the clinical trial period, Celecoxib (Celebrex® 100 mg capsules) was permitted as rescue medication for pain management at a maximum daily dose of 200 mg, administered either as a single 200 mg dose or 100 mg twice daily. However, to ensure accurate efficacy assessments, the administration of rescue medications was discontinued at least 5 days before the scheduled evaluation visit. In addition, the use of non-steroidal anti-inflammatory drugs outside the permitted concomitant drugs, including analgesics, systemic corticosteroids, immunosuppressants, muscle relaxants, and IA therapies, was prohibited and could lead to participant discontinuation.

### OSCA mechanism of action and intervention

The therapeutic efficacy of the OSCA intervention is mediated through modulation of the joint microenvironment. By restoring the balance between catabolic and anabolic signaling pathways, OSCA promotes tissue homeostasis within the osteoarthritic joint. This biological effect is driven by three synergistic mechanisms: stimulation of chondrogenic differentiation, which facilitates endogenous cartilage repair, immunomodulation, resulting in the attenuation of inflammatory responses, and protective effect through the inhibition of apoptosis in joint-resident cell. Collectively, these processes contribute to structural preservation of articular cartilage and improvement in overall joint function (Supplementary Fig. 1).

The frozen osiramestrocel and CAM components were thawed immediately before administration and aseptically combined into a single syringe. The injection site was aseptically prepared, and the solution was administered into the knee joint via the superolateral aspect by an orthopedic specialist uninvolved in patient assessment. Following administration, patients were closely monitored for 30 min for any adverse events, including injection site reactions.

### Biomarker measurement and sample collection

Urinary CTX-I and CTX-II were measured using ELISA kits (IDS, AC-03F1R and AC-10F1R; Boldon, UK) and normalized to urinary creatinine. Serum levels of CTX-I (IDS, AC-02F1R; Boldon), CTX-II (USCNK, CEA686Hu; Wuhan, China), MMP-3 (R&D Systems, DMP300; Minneapolis, MN, USA), PIIANP (MyBioSource, MBS109368; San Diego, CA, USA), and COMP (IDS, AC-23F1R; Boldon) were quantified via ELISA. Serum IL-1β and tumor necrosis factor-alpha (TNF-α) were assessed using a MILLIPLEX MAP Human High Sensitivity T Cell Panel-Immunology Multiplex Assay (Merck, HSTCMAG-28SK; Darmstadt, Germany). Urine and blood samples were collected at baseline and weeks 1, 4, 12, and 24. Midstream first-morning urine was stored at $\le$−70 °C. Blood samples were left at room temperature for 30 min, then centrifuged at 3,000 rpm for 10 min, and the serum aliquots were stored at $\le$−70 °C until analysis.

### Accuracy assessment

In each analytical run, calibration samples were analyzed in duplicate, and accuracy (% recovery) was evaluated using the mean value. If accuracy exceeded the acceptance criteria, the individual value was used instead.

### Outcome measurement

The primary end point was to assess DLT and to determine maximum tolerated dose after a single IA OSCA injection in patients with knee osteoarthritis. Safety and tolerability were evaluated based on the incidence of DLT, treatment-emergent adverse events (TEAEs), adverse drug reactions, and serious adverse events across all cohorts. The maximum tolerated dose was determined by monitoring DLT frequency and severity within a dose-escalation design, with adverse events classified and graded as per the National Cancer Institute Common Terminology Criteria for Adverse Events (NCI-CTCAE ver.5.0)^24^.

Secondary end points assessed exploratory efficacy after administration of OSCA over 24 weeks based on changes in pain and function scores using the International Knee Documentation Committee (IKDC)^25^, Knee Injury and Osteoarthritis Outcome Score (KOOS)^26^, Western Ontario and McMaster Universities Osteoarthritis Index (WOMAC)^27^, and VAS^28^ indices. Higher IKDC and KOOS scores indicate better clinical outcomes, whereas lower WOMAC and VAS scores denote symptom relief. Additional analysis of clinical efficacy was conducted to identify responders and non-responders using prespecified minimal clinically important difference-based criteria; responders were defined as those with a high improvement in pain or function ($\ge$50% relative improvement and $\ge$20-point absolute change from baseline)^29^. In addition, joint structural changes were evaluated using MRI and radiography to investigate the potential disease-modifying effects of OSCA. The Magnetic Resonance Observation of Cartilage Repair Tissue (MOCART) 2.0 score^30^ and Whole-Organ Magnetic Resonance Imaging Score (WORMS)^31^ were used for MRI assessment, whereas the K&L grading was used to evaluate radiographs. MOCART and WORMS assessments were performed on 3T MR systems (Achieva and Ingenia, Philips Medical Systems, Eindhoven, The Netherlands) at the baseline and 24 weeks after a single IA OSCA injection. Supplementary Table 2 shows the detailed MRI sequence parameters. An increased MOCART score indicated cartilage repair, whereas a decreased WORMS score and K&L grade indicated improved joint structure. All MRI and X-ray images were uploaded to a centralized clinical trial imaging management system (Trial Informatics, Seoul, Korea) and blindly evaluated by two independent radiologists. If the two primary readers disagreed, an adjudicating radiologist (third independent reader) reviewed their assessment scores and selected the most accurate result without conducting a separate evaluation. Additionally, exploratory biomarker analyses were conducted over 24 weeks to investigate the biological effects of OSCA on cartilage degradation and inflammation.

### Statistical analysis

Continuous variables are reported as mean and standard deviation, whereas categorical variables are reported as frequency and percentage. All end point analyses were conducted based solely on observed data, with no imputation performed for missing values. After normality assessment, either the Kruskal–Wallis test or one-way analysis of variance was used for all cohort comparisons of continuous variables, whereas the Mann–Whitney U test or Tukey’s honestly significant difference was used for post hoc pairwise comparisons as appropriate. Categorical variables were analyzed using Pearson’s χ or Fisher’s exact test, and within-cohort changes from baseline to 24 weeks were analyzed using the Wilcoxon signed-rank test. To account for within-subject correlation owing to repeated measurements and to evaluate longitudinal changes over time, a linear mixed-effect model was utilized to assess cohort differences over the 24-week follow-up period. The model included patients as random effects and cohorts, visit time, and their interactions as fixed effects. Post hoc pairwise comparisons were conducted to identify significant differences between cohorts. For clinical interpretability, responder proportions were compared across cohorts using Fisher’s exact test, based on predefined clinically meaningful thresholds corresponding to the minimal clinically important difference for pain and function. Data were analyzed with SAS Version 9.4 (SAS Institute, Cary, NC, USA). A two-sided *P-*value <0.05 indicated statistical significance.

## Results

### Baseline characteristics

Twelve patients were sequentially assigned to one of three dose cohorts: low-dose (*n* = 3), mid-dose (*n* = 3), and high-dose (*n* = 6). During the 24-week follow-up period, two patients in the high-dose group discontinued participation — one due to prohibited concomitant medication use and one due to adverse events. Ultimately, 10 patients completed the study. Baseline demographic and clinical measures (age, sex, body mass index, pain, and function) were similar across all three groups without statistically significant differences. Table 1 presents the detailed baseline characteristics.

**Table 1:** Demographic and baseline characteristics of the three cohorts.

	Low-dose, *n* = 3	Mid-dose, *n* = 3	High-dose, *n* = 6	*P-*value
Cells injected, no.	2.5 × 10^7^	5.0 × 10^7^	1.0 × 10^8^	NA
Age (years)	48.33 $\pm$ 18.23	55.67 $\pm$ 14.29	57.17 $\pm$ 7.08	0.758
Sex, *n* (%)				0.318
Male	2 (66.67)	0	4 (66.67)	
Female	1 (33.33)	3 (100)	2 (33.33)	
Height (cm)	167.93 $\pm$ 6.19	161.10 $\pm$ 12.00	169.96 $\pm$ 5.37	0.404
Weight (kg)	75.66 $\pm$ 3.66	58.66 $\pm$ 1.51	73.35 $\pm$ 11.34	0.091
Body mass index, (kg/m^2^)	26.93 $\pm$ 2.84	22.83 $\pm$ 3.12	25.26 $\pm$ 2.74	0.3385
Disease duration (years)	3.32 $\pm$ 2.39	3.56 $\pm$ 1.99	7.70 $\pm$ 10.38	0.944
KL grade, *n* (%)				NS
Grade 2	1 (33.33)	1 (33.33)	3 (50)	
Grade 3	2 (66.67)	2 (66.67)	3 (50)	
Grade 4	0	0	0	
Baseline VAS	70.00 $\pm$ 7.00	77.00 $\pm$ 13.23	67.67 $\pm$ 13.46	0.468
Baseline WOMAC	44.33 $\pm$ 10.02	50.00 $\pm$ 17.35	37.33 $\pm$ 21.77	0.415
Baseline IKDC	37.55 $\pm$ 14.04	41.38 $\pm$ 11.32	36.21 $\pm$ 14.43	0.933
Baseline KOOS				
Pain	44.45 $\pm$ 4.81	44.44 $\pm$ 19.45	54.63 $\pm$ 21.71	0.414
Symptoms	53.57 $\pm$ 3.57	46.43 $\pm$ 19.89	54.17 $\pm$ 21.95	0.856
Activities of daily living	55.88 $\pm$ 8.19	49.02 $\pm$ 19.19	60.54 $\pm$ 23.88	0.509
Sports and recreation	21.67 $\pm$ 24.66	23.33 $\pm$ 20.82	22.50 $\pm$ 18.10	0.956
Quality of life	27.08 $\pm$ 21.95	25.00$\,\pm$ 0.00	31.25 $\pm$ 15.31	0.813

Data are presented as mean ± standard deviation unless noted otherwise. *IKDC* International Knee Documentation Committee; *KL* Kellgren–Lawrence; *KOOS* Knee Injury and Osteoarthritis Outcome Score; *NA* not applicable; *NS* not significant; *VAS* Visual Analog Scale; *WOMAC* Western Ontario and McMaster Universities Osteoarthritis Index.

### Safety and tolerability

IA administration of OSCA was well tolerated across all cohorts, showing a favorable safety profile (Table 2). No DLTs or TEAEs causing death were reported throughout the study. TEAEs occurred in 25.0% (3/12 patients), with a total of four events, mostly mild-to-moderate (grades 2–3). The reported TEAEs were nasopharyngitis (cohort 2; *n* = 1), dyspepsia (cohort 3; *n* = 1), knee swelling (cohort 3; *n* = 1), and transverse myelitis (cohort 3, *n* = 1). An adverse drug reaction (knee swelling) occurred in cohort 3 (8.3%) and resolved within 6 weeks. In addition, one SAE (acute transverse myelitis) occurred in cohort 3 (8.3%) but was unrelated to the investigational product. Two patients in three cohorts discontinued owing to protocol deviations, including prohibited medication use and adverse events that prevented continued participation.

**Table 2:** Summary of adverse events.

Outcome	Low-dose (*n* = 3) *n* (%) [event]	Mid-dose (*n* = 3) *n* (%) [event]	High-dose (*n* = 6) *n* (%) [event]	All doses (*n* = 12) *n* (%) [event]
TEAEs	0 (0) [0]	1 (33.33) [1]	2 (33.33) [3]	3 (25.00) [4]
Nasopharyngitis	0 (0) [0]	1 (33.33) [1]	0 (0) [0]	1 (8.33) [1]
Dyspepsia	0 (0) [0]	0 (0) [0]	1 (16.67) [1]	1 (8.33) [1]
Knee swelling	0 (0) [0]	0 (0) [0]	1 (16.67) [1]	1 (8.33) [1]
Transverse myelitis	0 (0) [0]	0 (0) [0]	1 (16.67) [1]	1 (8.33) [1]
ADRs	0 (0) [0]	0 (0) [0]	1 (16.67) [1]	1 (8.33) [1]
SAEs	0 (0) [0]	0 (0) [0]	1 (16.67) [1]	1 (8.33) [1]
SADRs	0 (0) [0]	0 (0) [0]	0 (0) [0]	0 (0) [0]
DLT occurrence	0 (0) [0]	0 (0) [0]	0 (0) [0]	0 (0) [0]
TEAEs leading to death	0 (0) [0]	0 (0) [0]	0 (0) [0]	0 (0) [0]
TEAEs by severity				
Grade 1	0 (0) [0]	0 (0) [0]	0 (0) [0]	0 (0) [0]
Grade 2	0 (0) [0]	1 (33.33) [1]	2 (33.33) [2]	3 (25.00) [3]
Grade 3	0 (0) [0]	0 (0) [0]	1 (16.66) [1]	1 (8.33) [1]
Grade 4	0 (0) [0]	0 (0) [0]	0 (0) [0]	0 (0) [0]
Grade 5	0 (0) [0]	0 (0) [0]	0 (0) [0]	0 (0) [0]
TEAEs by relationship to IP				
Related	0 (0) [0]	0 (0) [0]	1 (16.67) [1]	1 (8.33) [1]
Not related	0 (0) [0]	1 (33.33) [1]	2 (33.33) [2]	3 (25.00) [3]
Results of TEAEs				
Recovered/resolved	0 (0) [0]	1 (33.33) [1]	2 (33.33) [2]	3 (25.00) [3]
Recovering/resolving	0 (0) [0]	0 (0) [0]	1 (16.66) [1]	1 (8.33) [1]
Not recovered/not resolved	0 (0) [0]	0 (0) [0]	0 (0) [0]	0 (0) [0]
Death	0 (0) [0]	0 (0) [0]	0 (0) [0]	0 (0) [0]
Unknown	0 (0) [0]	0 (0) [0]	0 (0) [0]	0 (0) [0]

*ADR* adverse drug reaction; *DLT* dose-limiting toxicity; *IP* investigational product; *SADR* serious adverse drug reaction; *SAE* serious adverse event; *TEAE* treatment-emergent adverse event.

### Clinical outcomes for pain and function

**Fig. 2:**
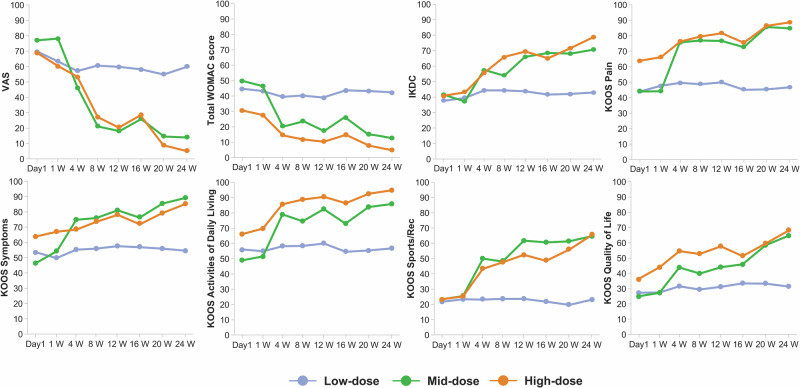
Clinical outcomes for pain and functional improvement following intra-articular administration of OSCA in knee osteoarthritis. Mean changes over 24 weeks in Visual Analog Scale, total Western Ontario and McMaster Universities Osteoarthritis Index score, International Knee Documentation Committee, and Knee Injury and Osteoarthritis Outcome Score subscales (pain, symptoms, activities of daily living, sports and recreation function, and quality of life) for patients receiving low-dose (blue), mid-dose (green), or high-dose (orange) OSCA.

IKDC scores increased from baseline (cohort 1: 37.55 $\pm$ 14.04; cohort 2: 41.38 $\pm$ 11.32; cohort 3: 40.52 $\pm$ 8.63) to 24 weeks (cohort 1: 42.91 $\pm$ 14.55; cohort 2: 70.88 $\pm$ 17.45; cohort 3: 79.02 $\pm$ 13.61), with mean changes of $+$5.36, $+$29.50, and $+$38.50 scores, corresponding to mean change rates of $+$15.3%, $+$89.7%, and $+$102.7%, respectively. WOMAC scores decreased from baseline (cohort 1: 44.33 $\pm$ 10.02; cohort 2: 50.00 $\pm$ 17.35; cohort 3: 31.00 $\pm$ 13.37) to 24 weeks (cohort 1: 42.00 $\pm$ 8.19; cohort 2: 13.67 $\pm$ 15.82; cohort 3: 5.00 $\pm$ 4.97), with mean changes of $-$2.33, $-$36.33, and $-$26.00 scores (mean change rates of $-$4.7%, $-$60.5%, and $-$76.2%, respectively). Similarly, VAS scores also decreased from baseline (cohort 1: 70.00 $\pm$ 7.00; cohort 2: 77.00 $\pm$ 13.23; cohort 3: 69.25 $\pm$ 13.05) to 24 weeks (cohort 1: 59.67 $\pm$ 8.14; cohort 2: 14.00 $\pm$ 17.06; cohort 3: 5.25 $\pm$ 5.50), with mean changes of $-$10.33, $-$63.00, and $-$64.00 scores (mean change rates of $-$14.6%, $-$78.9%, and $-$91.3%, respectively). All KOOS subscales, including pain, symptoms, stiffness, activities of daily living, sport and recreation function, and quality of life, improved across cohorts. Detailed numerical results for secondary end points are provided in Table 3 and Fig. 2.

**Table 3:** Clinical outcomes of VAS, WOMAC, IKDC, and KOOS for pain and function.

	Time	Low-dose, *n* = 3	Mid-dose, *n* = 3	High-dose, *n* = 4	*P-*value^a^
VAS	Baseline	70.00 ± 7.00	77.00 ± 13.23	69.25 ± 13.05	0.5691
	1 week	63.33 ± 7.51 (−9.34 ± 8.53)	78.33 ± 23.35 (0.17 ± 14.18)	60.50 ± 28.20 (−16.73 ± 30.70)	0.583
	4 weeks	57.33 ± 13.65 (−18.22 ± 15.80)	46.33 ± 39.50 (−39.47 ± 46.68)	53.25 ± 15.90 (−22.19 ± 20.89)	0.754
	8 weeks	60.67 ± 9.02 (−13.53 ± 6.01)	21.33 ± 22.19 (−68.30 ± 37.42)	27.25 ± 17.52 (−55.27 ± 39.19)	0.0626
	12 weeks	59.67 ± 4.04 (−14.51 ± 4.75)	17.67 ± 20.43 (−73.29 ± 34.30)	20.50 ± 10.08 (−67.03 ± 24.27)	0.0502
	16 weeks	58.00 ± 1.00 (−16.54 ± 9.07)	25.67 ± 16.74 (−63.17 ± 30.97)	28.00 ± 29.47 (−51.24 ± 61.79)	0.2293
	20 weeks	55.00 ± 3.61 (−21.23 ± 3.04)	14.67 ± 16.17 (−78.20 ± 26.72)	8.75 ± 6.13 (−86.16 ± 10.93)	0.0571
	24 weeks	59.67 ± 8.14 (−14.57 ± 10.03)	14.00 ± 17.06 (−78.81 ± 28.22)	5.25 ± 5.50 (−91.28 ± 9.58)	0.0546
	*P*-value^b^	0.109	0.109	0.068	
	LMM^c^ (Δ Change)	53.67 (16.63)			0.0022^†^
	LMM^c^ (% Change)	76.71 (20.05)			0.0004^†^
WOMAC	Baseline	44.33 ± 10.02	50.00 ± 17.35	31.00 ± 13.37	0.2152
	1 week	43.00 ± 9.64 (−2.83 ± 6.89)	46.67 ± 11.50 (1.89 ± 44.76)	27.75 ± 14.38 (−14.52 ± 17.46)	0.2231
	4 weeks	39.33 ± 5.69 (−9.99 ± 8.66)	20.33 ± 14.57 (−47.30 ± 52.03)	14.75 ± 16.26 (−52.31 ± 37.70)	0.1494
	8 weeks	40.00 ± 8.19 (−8.87 ± 10.76)	23.33 ± 19.60 (−37.16 ± 70.25)	12.00 ± 11.31 (−56.60 ± 35.93)	0.0874
	12 weeks	38.67 ± 8.96 (−11.65 ± 15.45)	17.67 ± 14.74 (−54.11 ± 46.09)	10.50 ± 9.95 (−60.62 ± 35.67)	0.0543
	16 weeks	43.33 ± 8.96 (−1.92 ± 3.33)	25.67 ± 14.57 (−34.70 ± 61.39)	15.00 ± 14.31 (−27.06 ± 99.67)	0.084
	20 weeks	43.00 ± 8.66 (−2.56 ± 4.44)	15.33 ± 11.24 (−60.47 ± 38.39)	8.00 ± 8.45 (−61.10 ± 55.45)	0.0364^*^
	24 weeks	42.00 ± 8.19 (−4.70 ± 4.27)	13.67 ± 15.82 (−60.49 ± 53.20)	5.00 ± 4.97 (−76.24 ± 33.82)	0.0522
	*P*-value^b^	0.18	0.18	0.068	
	LMM^c^ (Δ Change)	34.00 (15.105)			0.0289^*^
	LMM^c^ (% Change)	71.53 (31.63)			0.0282^†^
IKDC	Baseline	37.55 ± 14.04	41.38 ± 11.32	40.52 ± 8.63	0.9426
	1 week	39.47 ± 10.68 (7.92 ± 16.05)	37.17 ± 7.65 (−8.43 ± 12.44)	42.82 ± 6.53 (7.10 ± 13.31)	0.4455
	4 weeks	44.06 ± 16.31 (17.37 ± 17.03)	57.09 ± 8.93 (49.10 ± 65.17)	55.46 ± 15.77 (39.56 ± 45.61)	0.3775
	8 weeks	44.06 ± 17.97 (16.23 ± 6.18)	54.02 ± 16.29 (46.57 ± 91.27)	66.09 ± 13.22 (66.86 ± 40.48)	0.1999
	12 weeks	43.30 ± 16.10 (15.16 ± 7.36)	66.67 ± 15.47 (78.40 ± 98.35)	69.54 ± 7.99 (76.14 ± 33.49)	0.1469
	16 weeks	41.38 ± 10.96 (13.12 ± 13.49)	68.58 ± 13.86 (81.03 ± 89.60)	64.66 ± 15.94 (67.26 ± 57.12)	0.0899
	20 weeks	41.76 ± 11.51 (13.85 ± 12.26)	68.20 ± 15.61 (82.97 ± 101.51)	71.27 ± 15.13 (82.96 ± 55.73)	0.0553
	24 weeks	42.91 ± 14.55 (15.25 ± 3.86)	70.88 ± 17.45 (89.72 ± 106.02)	79.02 ± 13.61 (102.69 ± 54.59)	0.0777
	*P*-value^b^	0.102	0.109	0.068	
	LMM^c^ (Δ Change)	33.138 (11.48)			0.0058^†^
	LMM^c^ (% Change)	87.447 (39.40)			0.0311^†^
KOOS (pain)	Baseline	44.45 ± 4.81	44.44 ± 19.45	63.89 ± 13.80	0.1513
	1 week	48.15 ± 1.61 (8.88 ± 7.69)	44.45 ± 12.11 (5.79 ± 20.65)	66.67 ± 16.51 (3.80 ± 4.43)	0.2149
	4 weeks	50.00 ± 2.78 (13.70 ± 17.01)	75.93 ± 13.70 (109.10 ± 138.92)	76.39 ± 19.45 (19.24 ± 19.07)	0.1734
	8 weeks	49.08 ± 6.99 (11.11 ± 19.25)	76.85 ± 17.86 (115.05 ± 153.15)	79.86 ± 14.59 (26.44 ± 17.76)	0.0561
	12 weeks	50.00 ± 8.33 (13.33 ± 23.08)	76.85 ± 15.30 (112.79 ± 145.21)	81.95 ± 10.52 (30.85 ± 18.96)	0.0546
	16 weeks	45.37 ± 4.24 (2.22 ± 3.84)	73.15 ± 6.99 (96.37 ± 112.82)	75.70 ± 17.48 (23.37 ± 36.91)	0.0556
	20 weeks	45.37 ± 4.24 (2.22 ± 3.84)	86.11 ± 9.62 (130.12 ± 126.34)	86.81 ± 10.73 (41.02 ± 33.55)	0.0512
	24 weeks	46.30 ± 6.41 (4.44 ± 13.87)	85.19 ± 16.97 (134.79 ± 150.86)	88.89 ± 8.18 (43.60 ± 28.68)	0.0561
	*P*-value^b^	0.655	0.109	0.068	
	LMM^c^ (Δ Change)	38.89 (14.72)			0.0110*
	LMM^c^ (% Change)	130.35 (54.28)			0.0202*
KOOS (symptoms)	Baseline	53.57 ± 3.57	46.43 ± 19.89	64.29 ± 17.00	0.2982
	1 week	50.00 ± 9.45 (−6.51 ± 17.81)	54.76 ± 17.98 (22.74 ± 17.48)	66.97 ± 17.09 (4.65 ± 5.37)	0.3004
	4 weeks	55.95 ± 5.45 (4.92 ± 14.67)	75.00 ± 9.45 (95.23 ± 118.95)	68.75 ± 22.08 (6.58 ± 17.58)	0.2234
	8 weeks	55.95 ± 2.06 (4.76 ± 8.24)	76.19 ± 7.43 (97.08 ± 116.75)	74.11 ± 13.79 (21.42 ± 34.95)	0.0546
	12 weeks	58.33 ± 2.06 (9.20 ± 7.98)	80.95 ± 18.33 (117.19 ± 150.94)	78.57 ± 10.51 (28.45 ± 33.03)	0.0709
	16 weeks	57.14 ± 3.57 (7.14 ± 12.37)	76.19 ± 16.88 (103.44 ± 138.36)	72.32 ± 20.07 (20.25 ± 46.72)	0.3775
	20 weeks	55.95 ± 2.06 (4.76 ± 8.24)	85.71 ± 10.72 (117.45 ± 115.08)	79.47 ± 14.40 (30.98 ± 40.96)	0.0475*
	24 weeks	54.76 ± 4.12 (2.54 ± 10.70)	89.29 ± 12.37 (131.75 ± 138.68)	85.72 ± 9.22 (42.55 ± 47.56)	0.0475*
	*P*-value^b^	0.655	0.109	0.109	
	LMM^c^ (Δ Change)	41.67 (14.82)			0.0071*
	LMM^c^ (% Change)	129.21 (52.70)			0.0178*
KOOS (activities of daily living)	Baseline	55.88 ± 8.19	49.02 ± 19.19	65.81 ± 16.21	0.3162
	1 week	54.90 ± 9.79 (−1.84 ± 7.78)	51.47 ± 14.92 (13.48 ± 44.94)	69.85 ± 16.18 (6.66 ± 6.06)	0.2231
	4 weeks	58.33 ± 6.63 (4.72 ± 4.21)	78.43 ± 13.98 (82.79 ± 91.14)	85.66 ± 17.04 (35.41 ± 42.92)	0.1064
	8 weeks	58.33 ± 8.62 (4.50 ± 7.80)	74.02 ± 21.83 (76.99 ± 101.87)	88.97 ± 12.85 (41.25 ± 41.24)	0.0887
	12 weeks	59.81 ± 9.79 (7.21 ± 12.49)	81.86 ± 14.73 (90.21 ± 96.13)	90.08 ± 11.79 (43.29 ± 42.00)	0.0466*
	16 weeks	54.41 ± 8.95 (−2.70 ± 4.68)	72.55 ± 17.54 (71.42 ± 91.30)	86.03 ± 14.23 (39.31 ± 49.92)	0.0639
	20 weeks	54.90 ± 8.49 (−1.69 ± 5.76)	83.82 ± 13.07 (93.90 ± 93.82)	92.28 ± 8.18 (48.09 ± 44.65)	0.0371*
	24 weeks	56.37 ± 7.55 (1.01 ± 1.75)	85.30 ± 17.34 (99.93 ± 101.37)	94.49 ± 5.68 (51.06 ± 41.56)	0.0522
	*P*-value^b^	0.317	0.285	0.068	
	LMM^c^ (Δ Change)	35.79 (17.07)			0.0413*
	LMM^c^ (% Change)	98.92 (42.27)			0.0414*
KOOS (sports and recreation function)	Baseline	21.67 ± 24.66	23.33 ± 20.82	23.75 ± 16.52	0.992
	1 week	23.33 ± 18.93 (63.33 ± 118.46)	23.33 ± 20.21 (2.08 ± 20.62)	25.00 ± 16.83 (6.67 ± 11.55)	0.9915
	4 weeks	23.33 ± 23.09 (33.33 ± 57.74)	50.00 ± 0.00 (45.83 ± 29.46)	43.75 ± 33.76 (95.24 ± 100.34)	0.4864
	8 weeks	23.33 ± 23.09 (33.33 ± 57.74)	48.33 ± 20.82 (39.58 ± 109.01)	47.50 ± 29.01 (100.00 ± 93.68)	0.322
	12 weeks	23.33 ± 23.09 (33.33 ± 57.74)	61.67 ± 23.09 (68.75 ± 114.90)	52.50 ± 26.30 (114.29 ± 75.59)	0.1289
	16 weeks	21.67 ± 20.21 (30.00 ± 60.83)	60.00 ± 25.98 (62.50 ± 123.74)	48.75 ± 28.10 (92.38 ± 124.42)	0.1906
	20 weeks	20.00 ± 21.79 (−3.33 ± 5.77)	61.67 ± 23.09 (68.75 ± 114.90)	56.25 ± 30.65 (122.86 ± 120.10)	0.2275
	24 weeks	23.33 ± 23.09 (33.33 ± 57.74)	65.00 ± 30.00 (52.08 ± 91.33)	66.25 ± 22.87 (148.57 ± 102.90)	0.1513
	*P*-value^b^	0.317	0.285	0.068	
	LMM^c^ (Δ Change)	40.83 (17.11)			0.0209^†^
	LMM^c^ (% Change)	115.24 (65.29)			0.0863
KOOS (quality of life)	Baseline	27.08 ± 21.95	25.00 ± 0.00	35.94 ± 11.83	0.1513
	1 week	27.08 ± 19.09 (4.17 ± 19.09)	27.08 ± 3.61 (8.33 ± 14.43)	43.75 ± 13.50 (23.81 ± 16.03)	0.2149
	4 weeks	31.25 ± 21.65 (20.83 ± 47.32)	43.75 ± 12.50 (75.00 ± 50.00)	54.69 ± 20.65 (49.40 ± 11.23)	0.1734
	8 weeks	29.17 ± 14.43 (41.67 ± 62.92)	39.58 ± 20.09 (58.33 ± 80.36)	53.13 ± 23.11 (44.64 ± 24.39)	0.0561
	12 weeks	31.25 ± 16.54 (45.83 ± 56.37)	43.75 ± 27.24 (75.00 ± 108.97)	57.81 ± 17.21 (65.48 ± 27.87)	0.0546
	16 weeks	33.33 ± 13.01 (79.17 ± 109.21)	45.83 ± 25.26 (83.33 ± 101.04)	51.56 ± 24.14 (48.81 ± 57.59)	0.0556
	20 weeks	33.33 ± 13.01 (79.17 ± 109.21)	58.33 ± 25.26 (133.33 ± 101.04)	59.38 ± 18.75 (69.05 ± 27.36)	0.0512
	24 weeks	31.25 ± 10.83 (75.00 ± 114.56)	64.58 ± 31.46 (158.33 ± 125.83)	68.75 ± 15.31 (105.36 ± 66.34)	0.0561
	*P*-value^b^	0.564	0.109	0.068	
	LMM^c^ (Δ Change)	35.42 (11.83)			0.0043*
	LMM^c^ (% Change)	83.33 (53.31)			0.1244

Data are presented as mean ± standard deviation (change rate ± standard deviation). IKDC, International Knee Documentation Committee; KOOS, Knee Injury and Osteoarthritis Outcome Score; LMM, linear mixed model; VAS, Visual Analog Scale; WOMAC, Western Ontario and McMaster Universities Osteoarthritis Index. ^a^Kruskal–Wallis test was used to compare the differences in VAS, IKDC, WOMAC, and KOOS among the cohorts. ^b^Wilcoxon signed-rank test was used to compare the VAS, IKDC, WOMAC, and KOOS at baseline and visit 9 (24 weeks) in each cohort. ^c^LMM was used to detect differences among the cohorts during the 24 weeks and included patients as the random effect and cohorts, visit time, and their interaction as the fixed effects. Data are presented as least-squares mean difference (standard error). Symbols in the *P*-value column indicate statistically significant pairwise between-cohort differences (^*^cohort 1 versus cohort 2; ^†^cohort 1 versus cohort 3; *P* < 0.05); no statistically significant differences were observed between cohort 2 and cohort 3 (*P* > 0.05).

Following IA administration of OSCA, exploratory linear mixed-effect model analysis based on absolute change scores indicated that cohorts 2 and 3 showed numerically greater improvement in pain and functional outcomes over 24 weeks compared with cohort 1. Specifically, greater numerical improvements were observed in VAS (*P* = 0.002) and IKDC (*P* = 0.005) scores for cohort 3 and in WOMAC scores (*P* = 0.028) for cohort 2, compared with cohort 1. For the KOOS subscales, greater numerical improvements were noted in pain (*P* = 0.011), symptoms (*P* = 0.007), activities of daily living (*P* = 0.041), and quality of life (*P* = 0.004) for cohort 2 and in sports and recreation function (*P* = 0.021) for cohort 3, compared with cohort 1 (Table 3). The responder proportions, defined a priori using a clinically meaningful threshold, are summarized across VAS, WOMAC, IKDC, and KOOS subscales in Table 4. No responders were observed in the low-dose cohort across measures. By contrast, the high-dose cohort showed a 100% responder rate (4/4 patients) for the VAS, and a 75% rate (3/4 patients) was observed for the WOMAC, IKDC, and KOOS subscale (quality of life) (Table 4).

**Table 4:** Post hoc analysis of the number of patients achieving clinically meaningful pain or function improvement at 24 weeks.

	Low-dose, *n* = 3)	Mid-dose, *n* = 3	High-dose, *n* = 4	*P-* value
**VAS**				0.027
Responder (yes)	0 (0.0)	2 (66.7)	4 (100.0)	
Non-responder (no)	3 (100.0)	1 (33.3)	0 (0.0)	
**WOMAC**				0.115
Responder (yes)	0 (0.0)	2 (66.7)	3 (75.0)	
Non-responder (no)	3 (100.0)	1 (33.3)	1 (25.0)	
**IKDC**				0.129
Responder (yes)	0 (0.0)	1 (33.3)	3 (75.0)	
Non-responder (no)	3 (100.0)	2 (66.7)	1 (25.0)	
**KOOS (pain)**				0.217
Responder (yes)	0 (0.0)	2 (66.7)	2 (50.0)	
Non-responder (no)	3 (100.0)	1 (33.3)	2 (50.0)	
**KOOS (symptoms)**				0.197
Responder (yes)	0 (0.0)	2 (66.7)	1 (25.0)	
Non-responder (no)	3 (100.0)	1 (33.3)	3 (75.0)	
**KOOS** **(activities of daily living)**				0.217
Responder (yes)	0 (0.0)	2 (66.7)	2 (50.0)	
Non-responder (no)	3 (100.0)	1 (33.3)	2 (50.0)	
**KOOS** **(sports and recreation function)**				0.356
Responder (yes)	0 (0.0)	1 (33.3)	2 (50.0)	
Non-responder (no)	3 (100.0)	2 (66.7)	2 (50.0)	
**KOOS (quality of life)**				0.115
Responder (yes)	0 (0.0)	2 (66.7)	3 (75.0)	
Non-responder (no)	3 (100.0)	1 (33.3)	1 (25.0)	

A high improvement (responder) was defined as achieving a ≥50% relative Improvement and ≥20-point absolute change from baseline. *IKDC* International Knee Documentation Committee; *KOOS* Knee Injury and Osteoarthritis Outcome Score; *VAS* Visual Analog Scale; *WOMAC* Western Ontario and McMaster Universities Osteoarthritis Index.

### Radiological outcomes for joint structural changes

Across OSCA-treated cohorts, the mean MOCART score increased over 24 weeks, with seven patients (70%) showing improvement; however, this increase was not statistically significant (*P* > 0.05) (Supplementary Table 3).

**Fig. 3:**
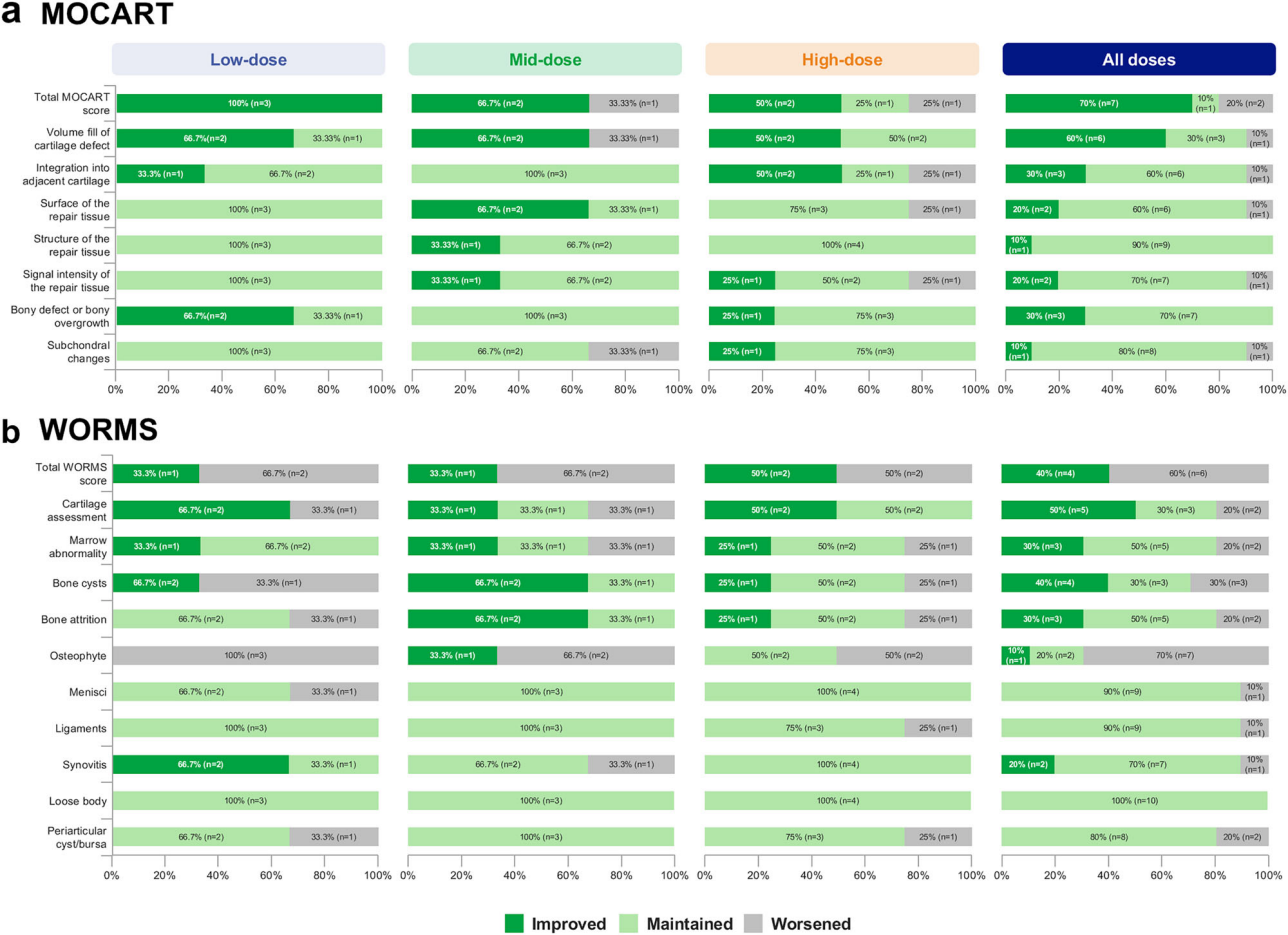
MRI assessment of structural changes using MOCART and WORMS scores across cohorts. **a** Percentage distribution of MOCART total scores and subscore changes at 24 weeks following intra-articular OSCA injection. Improvements (green), stability (light green), and worsening (grey). Across cohorts, 70% of patients showed improvement in MOCART scores, indicating potential cartilage repair effects. **b** WORMS total scores and subcomponent changes at 24 weeks across cohorts, with improvements (green), stability (light green), and worsening (grey). WORMS total score improved in 40% of patients, and cartilage integrity improved in 50% based on cartilage assessment subscores. Bone marrow abnormality scores were stable or improved in most participants, suggesting potential joint preservation effects of OSCA. MOCART, Magnetic Resonance Observation of Cartilage Repair Tissue; WORMS, Whole-Organ Magnetic Resonance Imaging Score.

**Fig. 4:**
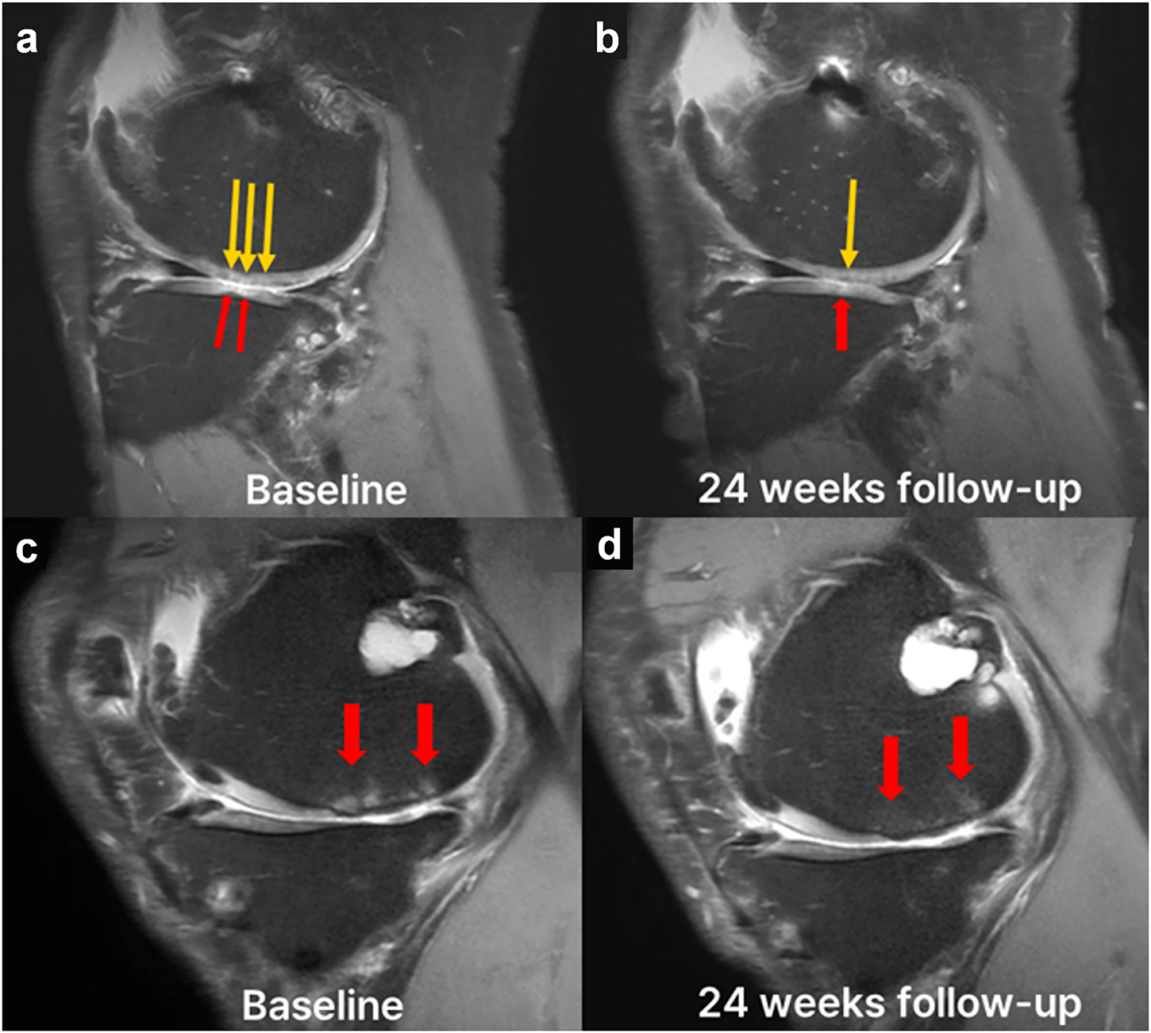
MRI assessment of cartilage repair and subchondral bone changes at baseline and 24 weeks post-OSCA administration. **a** Sagittal MRI at baseline shows cartilage defects in the lateral femoral condyle (yellow arrow) and lateral tibial plateau (red arrow). **b** At 24 weeks, MRI revealed increased cartilage coverage in the lateral femoral condyle (yellow arrow) and lateral tibial plateau (red arrow) with surface smoothening. **c** Baseline sagittal MRI images show subchondral bone marrow edema (red arrows) in the medial femoral condyle. **d** At 24 weeks, subchondral bone marrow integrity improved (red arrow).

Nine out of 10 (90%) patients maintained structural stability in subchondral bone; 8 (80%) showed no structural progression, and 1 (10%) improved (Fig. 3). Subchondral bone remodeling was observed exclusively in cohort 3, affecting one of the four patients (25%), with evidence of improved bone marrow and structural modification (Fig. 4). Additionally, cartilage defect volume fill improved in six patients (60%) across all cohorts.

Although the mean WORMS score showed no statistically significant changes from baseline to 24 weeks (Supplementary Table 3), four patients (40%) showed a reduction in WORMS total score. Five patients (50%) showed a reduction in the cartilage assessment component of WORMS (Figs. 3 and 4). However, osteophyte score worsened in seven patients (70%). Among the 10 participants, 2 (20%) exhibited a progression in K&L grade (one from grade 2 to grade 3 and one from grade 3 to grade 4), whereas 8 (80%) remained unchanged.

### Biomarker outcomes

Following OSCA administration, inflammation-related biomarkers tended to decrease over 24 weeks; however, none of the within-groups change from baseline to 24 weeks reached statistical significance. Serum IL-1β and TNF-α levels showed a downward trend over 24 weeks in the low-dose and medium-dose groups. Serum PIIANP tended to increase in the medium-dose and high-dose groups but decrease in the low-dose group, with no significant changes. Urinary CTX-II remained stable, whereas serum CTX-II showed a gradual, non-significant increase. Serum COMP and MMP-3 remained stable across all groups over 24 weeks (Supplementary Fig. 2).

## Discussion

This first-in-human, multicenter phase I study suggested that IA administration of OSCA might be a potential and innovative treatment for knee osteoarthritis, demonstrating a favorable efficacy and well-tolerated safety profile in low-dose, mid-dose, and high-dose of OSCA. In addition to symptomatic relief, the inclusion of both patient-reported outcomes and MRI-based scoring systems, such as MOCART and WORMS, allowed for a more structured evaluation of treatment effects on joint integrity. With respect to clinical relevance, structural changes on MRI such as cartilage thickness typically require longer follow-up periods often on the order of 2 years to demonstrate robust between-group differences in osteoarthritis trials. For instance, the FORWARD randomized clinical trial of IA sprifermin, the primary structural end point (change in total femorotibial joint cartilage thickness measured by quantitative MRI) was assessed at 2 years^32^. Although our MRI assessment relied on semi-quantitative scoring rather than quantitative thickness, directional changes in cartilage-related components may be clinically relevant; however, this phase I study was not powered to determine imaging–symptom relationships. The observation of osteophyte progression in some participants highlights the heterogeneity of structural remodeling and the need for longer-term controlled studies to clarify clinical significance. Taken together, observed clinical improvements and imaging-based structural changes suggest that OSCA may exert effects beyond symptom control and potentially influence the underlying disease process. Longer-term follow-up is currently ongoing to further validate these findings.

Several randomized controlled trials have evaluated IA MSC injections using WOMAC and WORMS, but a meta-analysis revealed limited evidence for pain relief and functional improvement, as well as insufficient evidence for cartilage repair^33^. Studies report that UCB-MSC-HA implants improve VAS, WOMAC, and IKDC scores^10,34^, but their use remains limited to patients receiving adjunctive treatment with microfracture for ICRS grade IV cartilage defects and not in the general osteoarthritis population. To our knowledge, no therapy is currently approved as a DMOAD that regenerates cartilage and modifies joint structure, underscoring the unmet need in knee osteoarthritis treatment. Most regenerative approaches target localized defects^35,36^, whereas our phase I data suggest that OSCA may promote symptomatic improvement with variable structural findings on MRI, warranting confirmation in further controlled trials. A notable strength of OSCA lies in its combination therapy strategy, in which CAM serves as a supportive adjunct to novel approach designed to enhance therapeutic efficacy by augmenting the activity of MSCs and promoting their regenerative capacity^22,23^. The numerical improvement in the volume fill of cartilage defect subscores in MOCART following OSCA administration may be mainly attributable to the optimized microenvironment created by OSCA, which could facilitate cartilage regeneration. This component enhances the local microenvironment to promote stem cell chondrogenic differentiation and restores essential cartilage components, such as sulfated glycosaminoglycans and collagen, thereby contributing to effective tissue repair^17,22^. Cartilage tissue improvement after OSCA administration aligns with the findings of previous preclinical studies^22,23^, further supporting their translational relevance.

Serum IL-1β and TNF-α, pro-inflammatory cytokines related to joint inflammation and cartilage degradation^18,19^, showed a non-significant decreasing trend up to 24 weeks in the low-dose and mid-dose cohorts after OSCA administration, suggesting a possible reduction in inflammatory burden in this exploratory phase I study. This trend may be attributable to the immunomodulatory properties of MSCs and the supportive microenvironment provided by CAM, which together may modulate IA inflammation. Serum PIIANP, a marker of cartilage synthesis^20^, tended to increase in the mid-dose and high-dose groups; however, in the absence of statistical correlation with clinical outcomes, these findings should be interpreted cautiously and considered hypothesis-generating.

WORMS analysis revealed that some patients exhibited osteophyte formation following OSCA administration. IA injection of stem cells activates key growth factors, such as TGF-βs and bone morphometric proteins, that have pivotal roles in joint tissue regeneration^21,37,38^. Notably, both TGF-βs and bone morphometric proteins have also been implicated in osteophyte formation within the joint^39–41^. Therefore, the observed osteophyte changes after OSCA administration may reflect activation of joint remodeling processes rather than definitive disease progression. Given the exploratory nature of this phase I study and the limited follow-up duration, osteophyte changes should be interpreted with caution and warrant long-term monitoring to clarify their clinical significance.

This study has some limitations. First, the small sample size of only 12 participants limits statistical power, generalizability, and the interpretability of inferential statistics; therefore, efficacy signals from secondary outcomes should be interpreted cautiously as exploratory. Second, the 24-week follow-up limited the assessment of long-term efficacy and safety. To address this limitation, a 5-year long-term follow-up study (NCT06013306) is currently ongoing and is expected to provide more definitive data on the long-term efficacy and safety of OSCA. Third, the study population included only Korean patients, which may limit the generalizability of the results to other ethnic backgrounds, especially with regard to body mass index. Finally, our study was not designed as a randomized, double-blind trial, which may introduce assessment biases and limit the strength of our findings. Despite limitations, our phase I clinical trial study provides valuable preliminary evidence supporting the feasibility, safety, and potential therapeutic efficacy of IA OSCA administration for knee osteoarthritis.

Over 24 weeks, numerical changes in pain and function outcomes were observed, and responder analyses based on a clinically meaningful threshold provided clinically interpretable context, although between-cohort comparisons should be considered exploratory given the small cohort sizes. MRI-based assessments showed mixed findings; these results should be considered hypothesis-generating and require confirmation in larger randomized controlled trials. These early-phase findings warrant further evaluation of OSCA as a potential DMOAD for knee osteoarthritis. To this end, a phase IIa trial involving 108 patients has commenced to further evaluate the efficacy and safety of OSCA as part of broader, long-term, large-scale clinical investigations.

## Supplementary information

Supplementary Information
