# Supplementary material for: First-in-human and multicenter phase I study of OSCA therapy for knee osteoarthritis
Source: Exp Mol Med. 2026 May 1;58(5):1510–21. doi: 10.1038/s12276-026-01728-w (PMC13234111; doi:10.1038/s12276-026-01728-w)
Supplement: Supplementary file 1 — Supplementary Information [file 12276_2026_1728_MOESM1_ESM.pdf]

Supplementary Information

Supplement to: Suh DK, Kang KS, Yoon KH et al. First-in-Human and Multicenter Phase I study of OSCA Therapy for Knee Osteoarthritis

This appendix has been provided by the authors to give readers additional information about the work.

Supplementary Figure

Supplementary Figure 1. Mechanism of Action of OSCA

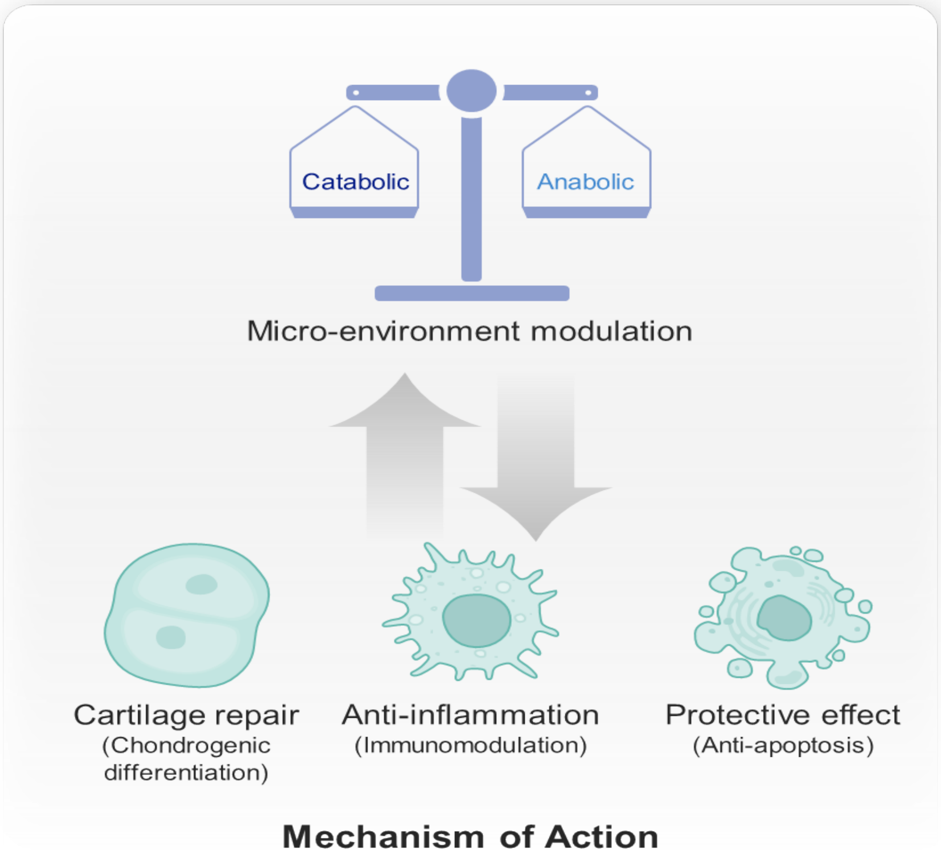

The figure illustrates the therapeutic mechanisms mediated by the modulation of the joint microenvironment. Through balancing catabolic and anabolic processes, the intervention exerts three primary effects: cartilage repair through chondrogenic differentiation, anti-inflammatory activity via immunomodulation, and cellular protection by inhibiting apoptosis.

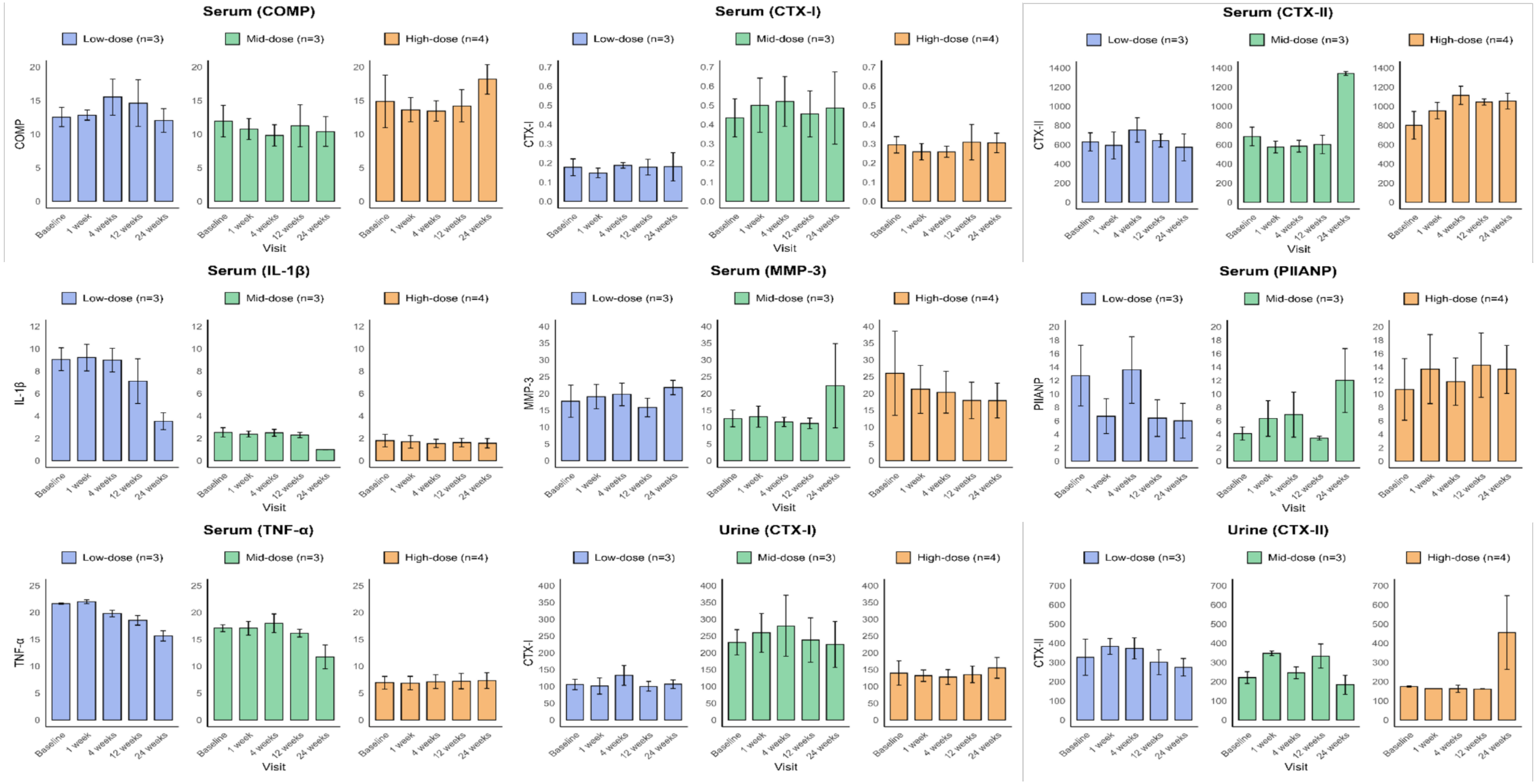

Supplementary Figure 2. Exploratory Biomarker Analysis of 24-Week OSCA Treatment

Serum and urine biomarkers associated with cartilage degradation and inflammation, including COMP (U/L), CTX-I (ng/mL) in serum; [µg/mmol] in urine) and CTX-II (pg/mL in serum; [ng/mmol] in urine), IL-1β (pg/mL), MMP-3 (ng/mL), PIIANP (ng/mL), and TNF-α (pg/mL) were assessed over the 24 weeks in low-dose, mid-dose, and high-dose OSCA treatment groups. Bars indicate mean values with standard errors. COMP, cartilage oligomeric matrix protein; CTX, C-terminal telopeptides of type I collagen; IL-1β, interleukin-1β; MMP-3, matrix metalloproteinase-3; PIIANP, procollagen type II N-terminal propeptide; TNF-α, tumor necrosis factor-α; CTX-II, C-terminal telopeptides of type II collagen.

Supplementary Table

Supplementary Table 1. Detailed Inclusion and Exclusion Criteria

Inclusion criteria

- Adult men and women aged ≥ 19 years
- Those with a body mass index (BMI) of < 30 kg/m^2 at screening
- Those classified as ICRS grade 3 or 4 according to the International Cartilage Repair Society (ICRS) rating system through MRI examination at the time of screening
- Those diagnosed with knee osteoarthritis according to the diagnosis criteria of the American College of Rheumatology (ACR) at the time of screening
- Those diagnosed with Kellgren–Lawrence (K&L) grade 2–3 knee osteoarthritis (OA) at the time of screening
- Those who do not have clinically significant abnormalities in the results of physical examination, hematologic test, blood chemistry test, or urine test
- Those whose symptoms (pain, and so on) do not improve despite conservative therapy (drug therapy, physical therapy, and so on) for at ≤ 12 weeks
- Those with a 100 mm Visual Analogue Scale (VAS) score for pain at the time of screening ≥ 50 mm
- Those who provided written informed consent after receiving and understanding the full trial details

Exclusion Criteria

- Those who have participated in at least one other clinical trial within 4 weeks before screening and were administered drugs (based on the last administration date), or were administered medical device treatment for clinical trials
- Those diagnosed with Kellgren–Lawrence (K&L) grade 4 knee OA at the time of screening
- At the time of screening, those with the results of the physical examination showing grade III (grade 0: none, grade I: 0–5 mm, grade II: 6–10 mm, grade III: > 10 mm) or more ligament instability
- Patients with osteochondritis dissecans
- Those with hypersensitivity reactions or allergies to components of the investigational product, OCSA Inj. (combination of (hUCB-MSCs) (solution 1) + (CAM Inj.) (solution 2))

- Those with the following confirmed medical history or surgical history/procedure history at the time of screening:
  - Patients with a history of procedure or surgery (including arthroscopy) on the target knee within 24 weeks before the baseline visit (Visit 2)
  - Patients who have undergone a knee joint replacement
- Those with any of the following diseases at the time of screening:
  - Active infectious disease (hepatitis A/B/C (including carriers) or HIV infection that, in the opinion of the investigator, renders those individuals unfit to participate in this clinical trial. However, those who test positive by vaccination may participate.)
  - Those who have been determined by the investigator as having difficulty participating in this clinical trial due to a severe chronic disease (cardiovascular disease, metabolic disease, renal dysfunction, and so on, excluding controlled hypertension)
  - Skin disease or infection at the intended injection site
  - Severe pain in other areas that may interfere with the knee symptom assessment (e.g., patients with poly-articular joint pain with severe OA symptoms in other areas (such as the hip joint))
  - Other joint diseases, such as inflammatory joint diseases, including rheumatoid arthritis, or infectious joint diseases such as septic arthritis, gout, recurrent pseudogout, joint fracture, acromegaly, Wilson's disease, primary osteochondrosis
  - Secondary OA due to systemic diseases (ochronosis or hemochromatosis)
  - Genetic disorders (hyperkinesia or collagen gene abnormalities)
  - Patients with severe painful conditions such as Sudeck's atrophy, Paget's disease, or spinal disc herniation
- Those with any of the following drug administration/treatment history:
  - Use of herbal drugs (for OA), glucosamine, chondroitin, anti-inflammatory drugs, patches, and/or other external preparations within 14 days before the baseline visit (Visit 2) (participation in the clinical trial may be allowed after a sufficient wash-out period, and for glucosamine and chondroitin, participation in clinical trials is possible for health functional foods only if

continuously administered from 4 weeks before screening)

- Those who have been given drugs through intra-articular injection A. Patients who were administered hyaluronic acid (HA) in the target joint cavity within 12 weeks of screening B. Patients who were administered steroids in the target joint cavity within 12 weeks of screening C. Patients who systemically used steroids within 4 weeks of screening (except for topical application and inhalation)
- Those who have performed physical therapy or oriental medical treatment (acupuncture, swelling, moxibustion, and so on) by a specialist for the purpose of relieving pain within 14 days before the baseline visit (Visit 2)
- Those who have been given an analgesic within 14 days before the administration of the investigational product, or within a time period five times the half-life of the analgesic.
- Those who have been given an immunosuppressive drug (cyclosporin A or azathioprine), including antirheumatic drugs (including methotrexate or antimetabolite) within 12 weeks before the baseline visit (Visit 2)
- Those who have been given an anesthetic within 2 days before the baseline visit (Visit 2)
- Gene therapy or cytotherapy for knee joint treatment

• Those who fall under the following laboratory test results at the time of screening:
  - Serum creatinine > 2.0 mg/dL renal dysfunction
  - AST or ALT test results > 2.5 times the upper limit of the normal range

• Patients with a malignant tumor (However, in the following cases, participation in the clinical trial may be allowed.)
  - Those who have been diagnosed with complete remission at least 5 years after completion of treatment for the tumor
  - If at least 1 year has elapsed since complete resection of a basal cell carcinoma/squamous cell carcinoma, radical resection of papillary thyroid cancer, or successful treatment of cervical epithelial cancer

• Women and men of reproductive potential who are pregnant, nursing, or unwilling to use appropriate contraception
  - Hormonal contraception (subcutaneous patch, injection, oral contraceptive,

amongst others.), intrauterine devices (loop, intrauterine system containing hormone), sterilization procedure for oneself or spouse (or partner) (vasectomy, tubal ligation, and so on.), double blocking (both male (condom) and female (contraceptive diaphragm, vaginal sponge or cervical cap) must use a contraceptive device), and so on.

- History of alcohol and drug abuse within 1 year before screening
- Any other condition deemed inappropriate for trial participation by the investigators

Supplementary Table 2. Detailed MRI Sequence Parameters

MRI Scan Parameters				
**Scanner Type**	1.5 Tesla or 3.0 Tesla			
**Patient Orientation**	Supine			
**Coil**	Dedicated knee coil			
**Sequences**	Proton Density FatSat TSE		T1 weighted imaging non-FatSat TSE	
**Magnetic field strength**	1.5 Tesla	3.0 Tesla	1.5 Tesla	3.0 Tesla
**Plane**	Sagittal & Coronal		Sagittal	
**Pulse Sequence**	TSE	TSE	TSE	TSE
**TR (msec)**	2800–4200	2800–4200	500–800	500–800
**TE (msec)**	20–40	20–40	10–20	10–20
**Field of View (cm)**	14–16	14–16	14–16	14–16
**Number of Slices**	25–30	25–30	25–30	25–30
**Slice Thickness (mm)**	3	3	3	3
**Slice Gap (%/mm)**	10%/0.3	10%/0.3	10%/0.3	10%/0.3
**NEX, NSA**	2	2	2	2
**Acquisition Matrix**	256–384 x 256–384	256-384 x 256-384	256–384 x 256–384	256–384 x 256–384
**Phase Encoding Direction**	Sagittal-AP Coronal-RL	Sagittal-AP Coronal-RL	AP	AP
**Flip Angle**	90–180	90–180	90	90

TSE, turbo spin echo; TR, repetition time; TE, echo time; NEX, number of excitations; NSA, number of signal averages; AP, anteroposterior; RL, right to left; MRI, magnetic resonance imaging.

Supplementary Table 3. Longitudinal Changes in MOCART and WORMS Score on MRI from baseline to follow-up (24 weeks)

	Categories	Time	(Low-dose, N = 3)	(Mid-dose, N = 3)	(High-dose, N = 4)	*p-value*[a]
**MOCART**	Total MOCART Score	Baseline	15.00 ± 5.00	37.50 ± 31.22	34.38 ±15.60	0.3329
		24 weeks	24.17 ± 9.46	45.83 ± 40.18	36.88 ± 6.88	0.4372
		Δ Change	9.17 ± 6.29	8.33 ± 9.46	2.50 ± 16.46	0.8066
		LMM[b]	6.67 (6.68)			0.3515
		p-value[c]	0.109	0.285	0.593	
	Volume fill of cartilage defect	Baseline	0.83 ± 1.44	5.83 ± 2.89	3.13 ± 2.39	0.1133
		24 weeks	2.50 ± 2.50	7.50 ± 6.61	5.63 ± 3.15	0.4215
		Δ Change	1.67 ± 1.44	1.67 ± 3.82	2.50 ± 3.54	0.9913
		LMM[b]	0.83 (1.71)			0.6424
		p-value[c]	0.157	0.414	0.18	
	Integration into the adjacent cartilage	Baseline	2.50 ± 4.33	8.33 ± 7.64	6.25 ± 4.79	0.3858
		24 weeks	3.33 ± 5.77	8.33 ± 7.64	5.63 ± 5.15	0.5552
		Δ Change	0.83 ± 1.44	0.00 ± 0.00	-0.63 ± 6.57	0.7391
		LMM[b]	0.83 ± 2.52			0.751
		p-value[c]	0.317	NS	NS	

	Surface of the repair tissue	Baseline	0.00 ± 0.00	0.00 (0.00)	1.25 ± 2.50	0.4724
		24 weeks	0.00 ± 0.00	1.67 (1.44)	0.00 ± 0.00	0.0724
		Δ Change	0.00 (0.00)	1.67 (1.44)	-1.25 ± 2.50	0.093
		LMM[b]	2.92 (0.98)			0.0204[‡]
		p-value[c]	NS	0.157	0.317	
	Structure of the repair tissue	Baseline	0.00 ± 0.00	0.00 ± 0.00	0.00 ± 0.00	NS
		24 weeks	0.00 ± 0.00	3.33 (5.77)	0.00 ± 0.00	0.3114
		Δ Change	0.00 ± 0.00	3.33 (5.77)	0.00 ± 0.00	0.3114
		LMM[b]	3.33 (1.67)			0.0856
		p-value[c]	NS	0.317	NS	
	Signal intensity of the repair tissue	Baseline	3.33 ± 5.77	3.33 ± 5.77	2.50 ± 5.00	0.9649
		24 weeks	3.33 ± 5.77	6.67 ± 5.77	2.50 ± 5.00	0.5523
		Δ Change	0.00 ± 0.00	3.33 ± 5.77	0.00 (8.16)	0.6981
		LMM[b]	3.33 (3.33)			0.3506
		p-value[c]	NS	0.317	NS	
	Bony defect or bony overgrowth	Baseline	0.83 ± 1.44	6.67 ± 5.77	7.50 ± 5.00	0.2553
		24 weeks	7.50 ± 4.33	6.67 ± 5.77	8.13 ± 3.75	0.8964

		Δ Change	6.67 ± 5.77	0.00 ± 0.00	0.63 ± 1.25	0.1621
		LMM[b]	6.67 (1.84)			0.0085*
		p-value[c]	0.157	NS	0.317	
	Subchondral changes	Baseline	7.50 ± 6.61	13.33 ± 11.55	13.75 ± 9.46	0.4235
		24 weeks	7.50 ± 6.61	11.67 ± 10.41	15.00 ± 10.00	0.3493
		Δ Change	0.00 ± 0.00	-1.67 ± 2.89	1.25 ± 2.50	0.2691
		LMM[b]	2.92 (1.21)			0.0474[‡]
		p-value[c]	NS	0.317	0.317	
	Total WORMS score	Baseline	83.33 ± 72.75	73.33 ± 44.88	61.38 ± 56.45	0.6889
		24 weeks	87.83 ± 74.69	69.33 ± 30.09	62.38 ± 57.16	0.6889
		Δ Change	4.50 ± 6.50	-4.00 ± 14.80	1.00 ± 2.45	0.7032
		LMM[b]	8.50 (5.07)			0.1378
		p-value[c]	0.285	NS	0.461	
	Cartilage Assessment	Baseline	39.67 ± 39.45	37.33 ± 27.13	24.00 ± 16.20	0.5463
		24 weeks	40.00 ± 38.46	38.83 ± 26.27	23.25 ± 15.84	0.6166
		Δ Change	0.33 ± 2.36	1.50 ± 3.04	-0.75 ± 0.87	0.4815
		LMM[b]	2.25 (1.15)			0.092

WORMS		*p-value*[c]	NS	0.655	0.157	
	Marrow Abnormality	Baseline	11.00 ± 10.15	5.50 (4.33)	4.38 (2.29)	0.7725
		24 weeks	10.33 ± 9.07	4.50 (2.18)	4.50 (2.74)	0.5849
		Δ Change	-0.67 (1.15)	-1.00 ± 2.18	0.13 (1.03)	0.7602
		LMM[b]	1.125 (0.80)			0.2024
		p-value[c]	0.317	0.655	0.655	
	Bone cysts	Baseline	8.50 ± 9.58	4.50 ± 1.80	2.13 ± 1.31	0.1835
		24 weeks	8.67 ± 10.02	3.17 ± 2.84	2.50 ± 1.58	0.6279
		Δ Change	0.17 ± 1.04	-1.33 ± 1.53	0.38 ± 1.49	0.3102
		LMM[b]	1.71 (0.75)			0.057
		p-value[c]	0.785	0.18	0.655	
	Bone Attrition	Baseline	2.50 ± 3.50	2.00 ± 2.65	1.13 ± 1.03	0.9658
		24 weeks	3.67 ± 5.51	0.00 ± 0.00	1.38 ± 2.14	0.2599
		Δ Change	1.17 ± 2.02	-2.00 (2.65)	0.25 ±1.26	0.1852
		LMM[b]	3.17 (1.13)			0.0266*
		p-value[c]	0.317	0.18	0.655	
	Osteophyte	Baseline	12.33 ± 7.25	17.00 ± 6.73	21.13 ± 30.26	0.6756

		24 weeks	15.67 ± 8.98	15.50 ± 4.33	21.75 ± 31.20	0.6825
		Δ Change	3.33 ± 3.01	-1.50 ± 8.89	0.63 ± 0.95	0.338
		LMM[b]	4.83 (2.92)			0.1417
		p-value[c]	0.109	NS	0.18	
	Menisci	Baseline	6.67 ± 3.06	5.00 ± 2.65	6.00 ± 3.92	0.7922
		24 weeks	7.00 ± 2.65	5.00 ± 2.65	6.00 ± 3.92	0.6756
		Δ Change	0.33 ± 0.58	0.00 ± 0.00	0.00 ± 0.00	0.3114
		LMM[b]	0.33 (0.17)			0.0856
		p-value[c]	0.317	NS	NS	
	Ligaments	Baseline	0.00 ± 0.00	0.00 ± 0.00	0.50 ± 1.00	0.4724
		24 weeks	0.00 ± 0.00	0.00 ± 0.00	0.63 ± 1.25	0.4724
		Δ Change	0.00 ± 0.00	0.00 ± 0.00	0.13 ± 0.25	0.4724
		LMM[b]	0.125 (0.089)			0.2002
		p-value[c]	NS	NS	0.317	
		Baseline	1.33 ± 0.29	1.00 ± 1.00	1.00 ± 0.71	0.8363
		24 weeks	0.83 ± 0.76	1.33 ± 0.58	1.00 ± 0.71	0.7788
	Synovitis	Δ Change	-0.50 ± 0.50	0.33 ± 0.58	0.00 ± 0.00	0.0852

		LMM[b]	0.83 ± 0.24			0.0095†
		p-value[c]	0.18	0.317	NS	
	Loose Body	Baseline	0.33 ± 0.58	0.00 ± 0.00	0.63 ± 1.25	0.6193
		24 weeks	0.33 ± 0.58	0.00 ± 0.00	0.63 ± 1.25	0.6193
		Δ Change	0.00 ± 0.00	0.00 ± 0.00	0.00 ± 0.00	>0.9999
		LMM[b]	NS			NS
		p-value[c]	NS	NS	NS	
	Periarticular Cyst/Bursa	Baseline	1.00 ± 0.00	1.00 ± 1.00	0.50 ± 0.58	0.4696
		24 weeks	1.33 ± 0.58	1.00 ± 1.00	0.75 ± 0.50	0.5188
		Δ Change	0.33 ± 0.58	0.00 ± 0.00	0.25 ± 0.50	0.5971
		LMM[b]	0.33 (0.26)			0.2402
		p-value[c]	0.317	NS	0.317	

Data are presented as mean ± standard deviation. [a]Kruskal–wallis test was used to compare the differences in MOCART and WORMS scores among the dose groups. [b]LMM was used to detect differences between the dose groups during the 24 weeks and included patients as the random effect and dose groups, visit time, and visit × dose groups interaction as the fixed effects. Data are presented as least squares mean difference (standard error). [c]Wilcoxon signed-rank test was used to compare the MOCART and WORMS sub-scores at baseline and 24 weeks in each dose group. Symbols(*,†,‡) signify p values between low-dose and mid-dose(*), low-dose and high-dose(†), and mid-dose and high-dose(‡). LMM, linear mixed model; MOCART, Magnetic Resonance Observation of Cartilage Repair Tissue; WORMS, Whole-Organ Magnetic Resonance Imaging Score; NS, not significant.
